# Medical emergency calls and calls for central nervous system symptoms during the COVID-19 outbreak in Hangzhou, China

**DOI:** 10.3389/fpubh.2022.934403

**Published:** 2022-11-24

**Authors:** Xinyan Fu, Chunyi Wang, Wen Wen, Jiake Tang, Chen Chen, Yongran Cheng, Mengyun Zhou, Qi Wu, Xingwei Zhang, Zhanhui Feng, Mingwei Wang, Ping Yu

**Affiliations:** ^1^Hangzhou Institute of Cardiovascular Diseases, Affiliated Hospital of Hangzhou Normal University, Hangzhou, China; ^2^Clinical School of Medicine, Hangzhou Normal University, Hangzhou, China; ^3^School of Public Health, Hangzhou Medical College, Hangzhou, China; ^4^Department of Molecular and Cellular Physiology, Shinshu University School of Medicine, Matsumoto, Japan; ^5^Department of Neurology, Affiliated Hospital of Guizhou Medical University, Guiyang, China

**Keywords:** medical emergency calls, central nervous system, COVID-19, emergency center, SARS-CoV-2

## Abstract

**Background:**

Since January 2020, the continuous and severe COVID-19 epidemic has ravaged various countries around the world and affected their emergency medical systems (EMS). The total number of emergency calls and the number of emergency calls for central nervous system (CNS) symptoms during the 2020 COVID-19 outbreak in Hangzhou, China (January 20–March 20) were investigated, and it was investigated whether these numbers had decreased as compared with the corresponding period in 2019.

**Methods:**

The number of daily emergency calls, ambulance dispatches, and rescues at the Hangzhou Emergency Center (HEC) was counted. The CNS symptoms considered in this study included those of cerebrovascular diseases, mental and behavioral disorders, and other neurological diseases.

**Results:**

It was found that, during the 2020 study period, the number of emergency calls was 33,563, a decrease of 19.83% (95% CI: 14.02–25.41%) as compared to the 41,863 emergency calls in 2019 (*P* < 0.01). The number of ambulances dispatched was 10,510, a decrease of 25.55% (95 %CI: 18.52–35.11%) as compared to the 14,117 ambulances dispatched in 2019 (*P* < 0.01). The number of rescues was 7,638, a decrease of 19.67% (95% CI: 16.12–23.18%) as compared with the 9,499 rescues in 2019 (*P* < 0.01). It was also found that the number of emergency calls related to CNS symptoms, including symptoms of cerebrovascular diseases, mental and behavioral disorders, and other neurological diseases, was significantly reduced (*P* < 0.01).

**Conclusion:**

The total number of medical emergency calls and the number of emergency calls for CNS symptoms occurring in a large city in China decreased significantly during the COVID-19 epidemic.

## Introduction

In December 2019, a case of pneumonia of unknown cause was reported in China. Since then, SARS-CoV-2 (commonly known as the “coronavirus” or COVID-19) has been declared a global pandemic (1). The peak of the epidemic in China occurred from January to March 2020, but the number of infections is currently increasing exponentially in the United States, India, and Brazil (2). During the epidemic, as a preventive measure, many hospitals in China opened fever clinics and shut down routine outpatient clinics, especially those for ophthalmology, otolaryngology, and stomatology, and encouraged people not to go to the hospital unless necessary. These measures have greatly reduced the number of patients seeking medical care due to non-COVID-related symptoms (3). A growing body of evidence indicates that people experiencing medical emergencies are avoiding the emergency department due to fear of contracting COVID-19, leading to increased morbidity and mortality (4). Recent reports have shown that the number of emergency room visits for cardiovascular diseases in the United States, Italy, and Hong Kong during the COVID-19 pandemic has declined (5–7). It should also be noted that winter and spring are characterized by a high incidence of cerebrovascular diseases (8). Since the start of the COVID-19 epidemic in China, the number of emergency calls, especially those related to CNS symptoms, seems to have decreased, which raises the question of why this change might have occurred. In this article, the possible reasons for this situation are discussed, and corresponding solutions are proposed. It is the hope of the authors that this research can provide a certain foundation for future public health undertakings.

The Hangzhou Emergency Center (HEC), an institution directly managed by the Hangzhou Municipal Government, undertakes pre-hospital first aid. During the COVID-19 epidemic in Hangzhou, a substantial increase in the number of calls for respiratory illnesses was not found; instead, a decrease was found in the number of calls for other illnesses for all types of patients (e.g., those in wartime, major natural disasters, and accidents) from all over the city. On a daily basis, the number of distress calls received in 2019 was between 600 and 750, but with the arrival of various holidays and flu season, the number of emergency calls will often increase.

**Figure 1:**
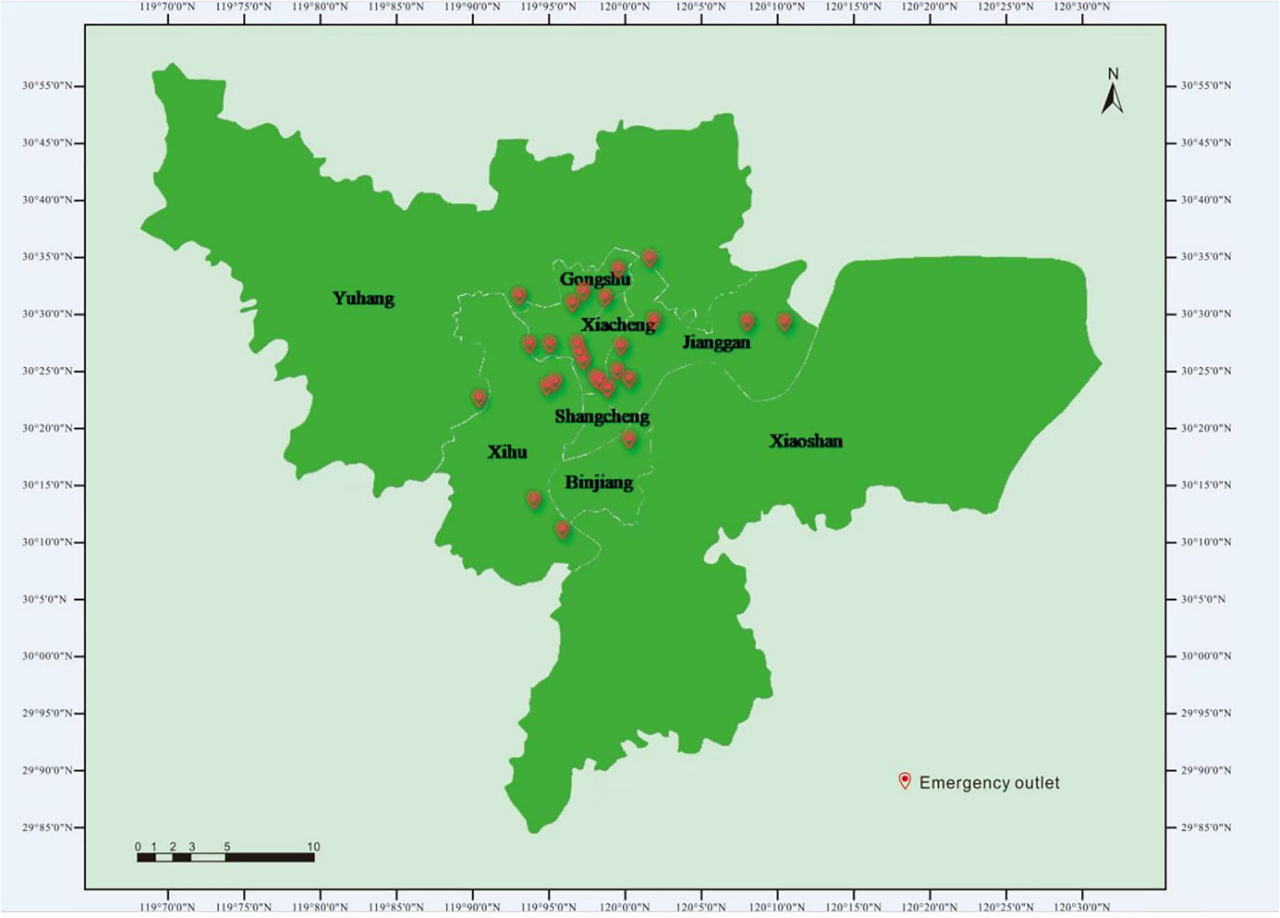
Hangzhou Emergency Center (HEC) radiation range and emergency outlet.

**Figure 2:**
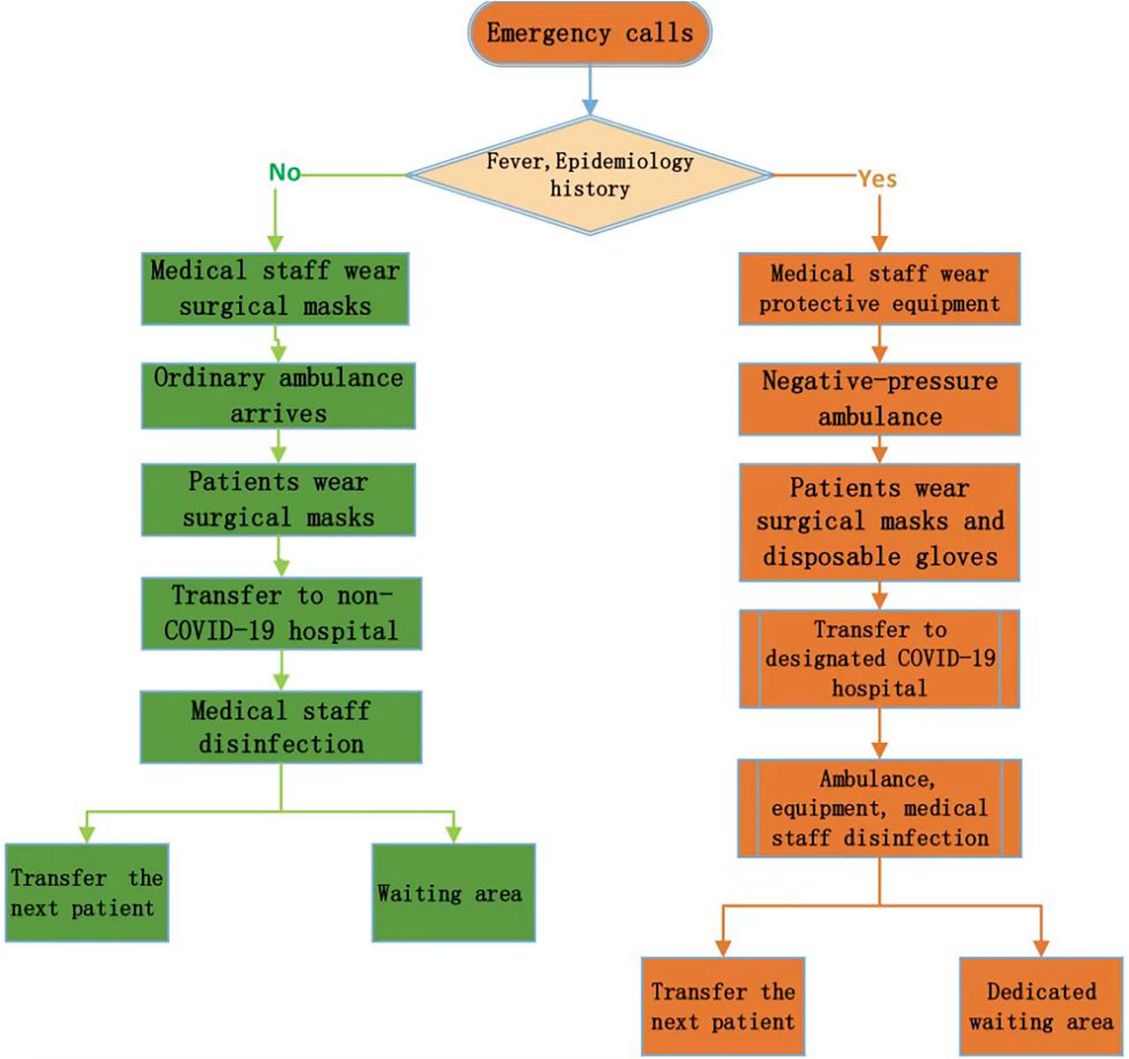
The transfer process of the HEC during the COVID-19 epidemic. Protective equipment includes disposable working caps, protective glasses, protective medical masks (N95), protective clothing, disposable latex gloves, and disposable shoe covers. After returning to the HEC, the negative-pressure ambulance is parked in a special decontamination area; the space inside the ambulance and stretchers, carts, and other items is disinfected with a 1:1 quaternary ammonium salt disinfectant; the doors and windows are closed for at least 30 min, and 30 min of UV light irradiation is performed. In addition, the medical staff does not remove protective clothing according to the professional process.

As of 15 March 2020, a total of 181 cases of COVID-19 had been reported in Hangzhou, and from then until August 1, there were no new cases (9). The incidence of COVID-19 in Hangzhou was 2.17/10,000, and the case fatality rate was 0 (10). Moreover, the incidence of pneumonia in Zhejiang Province was 2.34/10,000, and the case fatality rate was 0 (11). Hangzhou has two hospitals for the treatment of suspected and confirmed COVID-19 cases, as well as an intensive care center. Moreover, 10 public hospitals in Hangzhou have fever clinics, which are used to distinguish suspected patients with COVID-19 from ordinary patients and avoid cross-infection (12). The HEC includes 25 emergency outlets (Figure 1). During the epidemic, special procedures have been implemented for ambulances, and the emergency admission procedures for suspected patients and ordinary patients are different (Figure 2). By analyzing the number of medical emergency calls to the HEC during the epidemic, it was found that there was a significant change as compared to the same period in 2019. This study focuses on analyzing the change in the number of emergency calls for CNS symptoms, exploring the reasons for the decline in the number of emergency calls, and seeking solutions to prevent the populace from avoiding the use of the emergency medical system (EMS) in life-threatening situations.

## Methods

### Research object

The study period was from 20 January to 20 March 2020. The number of daily emergency calls, ambulance dispatches, and rescues of the HEC during this period was counted, as was the number of patients with CNS symptoms. Moreover, the corresponding data for the same period in 2019 were collected. The data from the HEC are provided to illustrate the downward trend in the number of emergency calls in recent months.

### Selection of the statistical time period

The first case of COVID-19 in Hangzhou appeared on January 19, and the HEC formulated a transfer plan on January 20. Therefore, January 20 was selected as the starting point for statistics. During the epidemic, Hangzhou residential areas were managed to ensure closures. Until March 15, all cases of COVID-19 had been cured, and the surrounding communities were gradually opened around March 20; therefore, March 20 was selected as the statistical end point. However, it currently remains necessary for the public to wear masks, take body temperature measurements, and display their health code when entering public places. The “first aid” function on the health code platform can be used to make emergency calls to the HEC, and this will later be discussed in detail.

### Nervous system symptoms

The operating staff of the HEC emergency system records the main and most serious complaints and then classifies the main complaints as clinical diagnoses. CNS symptoms include symptoms of cerebrovascular diseases, mental and behavioral disorders, and neurological diseases. Cerebrovascular diseases include cerebrovascular accidents, cerebral hemorrhage, subarachnoid hemorrhage, cerebral infarction, cerebral thrombosis, sequelae of cerebral vascular accidents, and cerebral aneurysms. Mental and behavioral disorders include hysteria, neurasthenia, depression, obsessive-compulsive disorder, anxiety, and schizophrenia. Other neurological diseases include encephalitis, headache, hemiplegia, aphasia, coma, meningitis, Parkinson's disease, epilepsy, transient ischemic attack, myasthenia gravis, and others.

### Statistical indicators

The statistical indicators were (1) comparisons between the number of emergency calls, ambulance dispatches, and rescues in 2019 and 2020, and (2) comparisons between the number of emergency calls for cerebrovascular diseases, mental and behavioral disorders, and other neurological diseases in 2019 and 2020.

#### Statistical analysis

The numbers and percentages of emergency calls, ambulance dispatches, and rescues during 2019 and 2020 were used for descriptive statistics, and descending rate and corresponding 95%CI are given, respectively. An independent sample *t*-test was conducted for statistical analysis. *P* < 0.05 (two-sided) were considered statistically significant. Statistical analyses were conducted using the *R* statistical software (version 3.6.1; http://www.Rproject.org).

## Results

### Comparison between the number of emergency calls, ambulance dispatches, and rescues in 2019 and 2020

**Figure 3:**
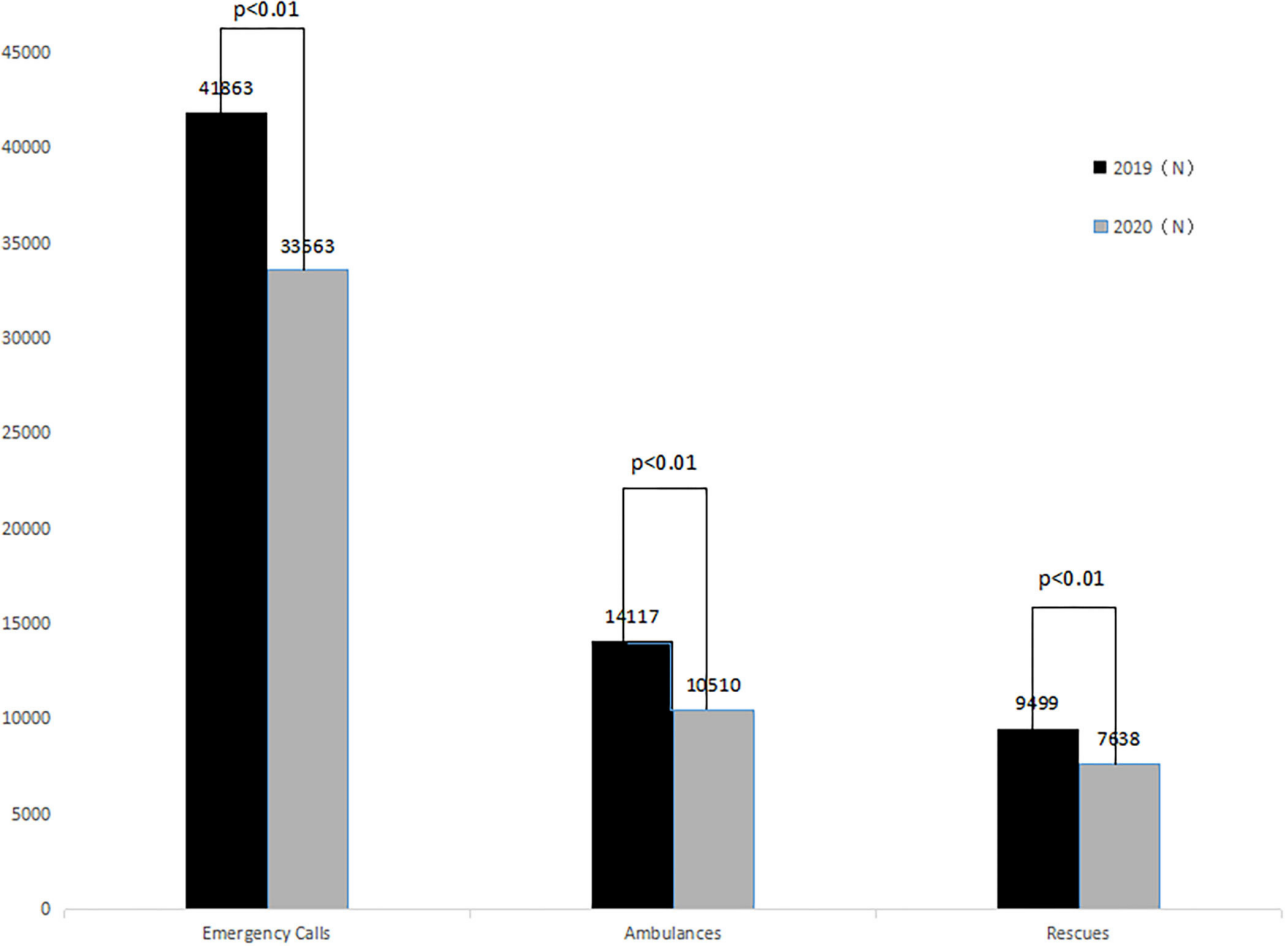
Comparison between the number of emergency calls, ambulance dispatches, and rescues in 2019 and 2020.

The total number of emergency calls during the COVID-19 epidemic in 2020 was 33,563, a decrease of 19.83% (95% CI: 14.02–25.41%) as compared to the 41,863 emergency calls during the same period in 2019 (*P* < 0.01). The number of ambulance dispatches was 10,510, a decrease of 25.55% (95% CI: 18.52–35.11%) as compared to the 14,117 ambulance dispatches during the same period in 2019 (*P* < 0.01). The number of rescues was 7,638, a decrease of 19.67% (95% CI: 16.12–23.18%) as compared to the 9,499 rescues during the same period in 2019 (*P* < 0.01). The *P*-values for these comparisons are all < 0.01, indicating that the differences are statistically significant. These data are presented in Figure 3.

### Comparison between the number of emergency calls, ambulance dispatches, and rescues per day in 2019 and 2020

**Figure 4:**
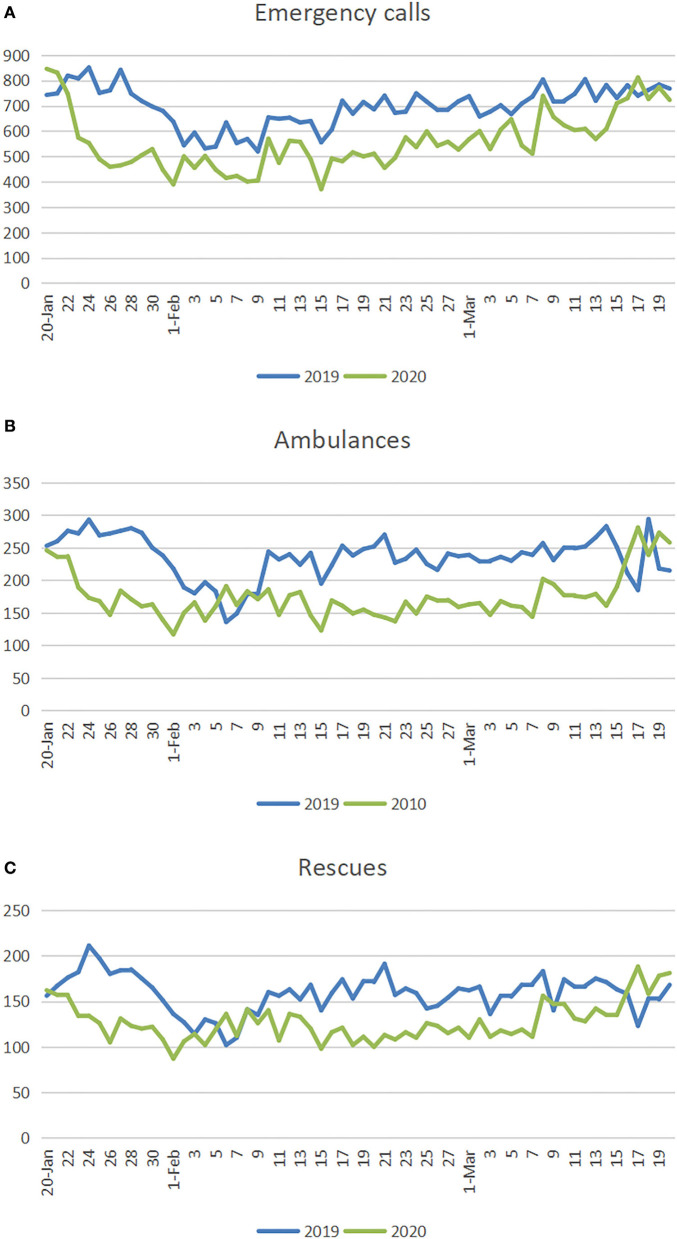
**(A–C)** Comparison between the number of emergency calls, ambulance dispatches, and rescues per day in 2019 and 2020.

**Figure 5:**
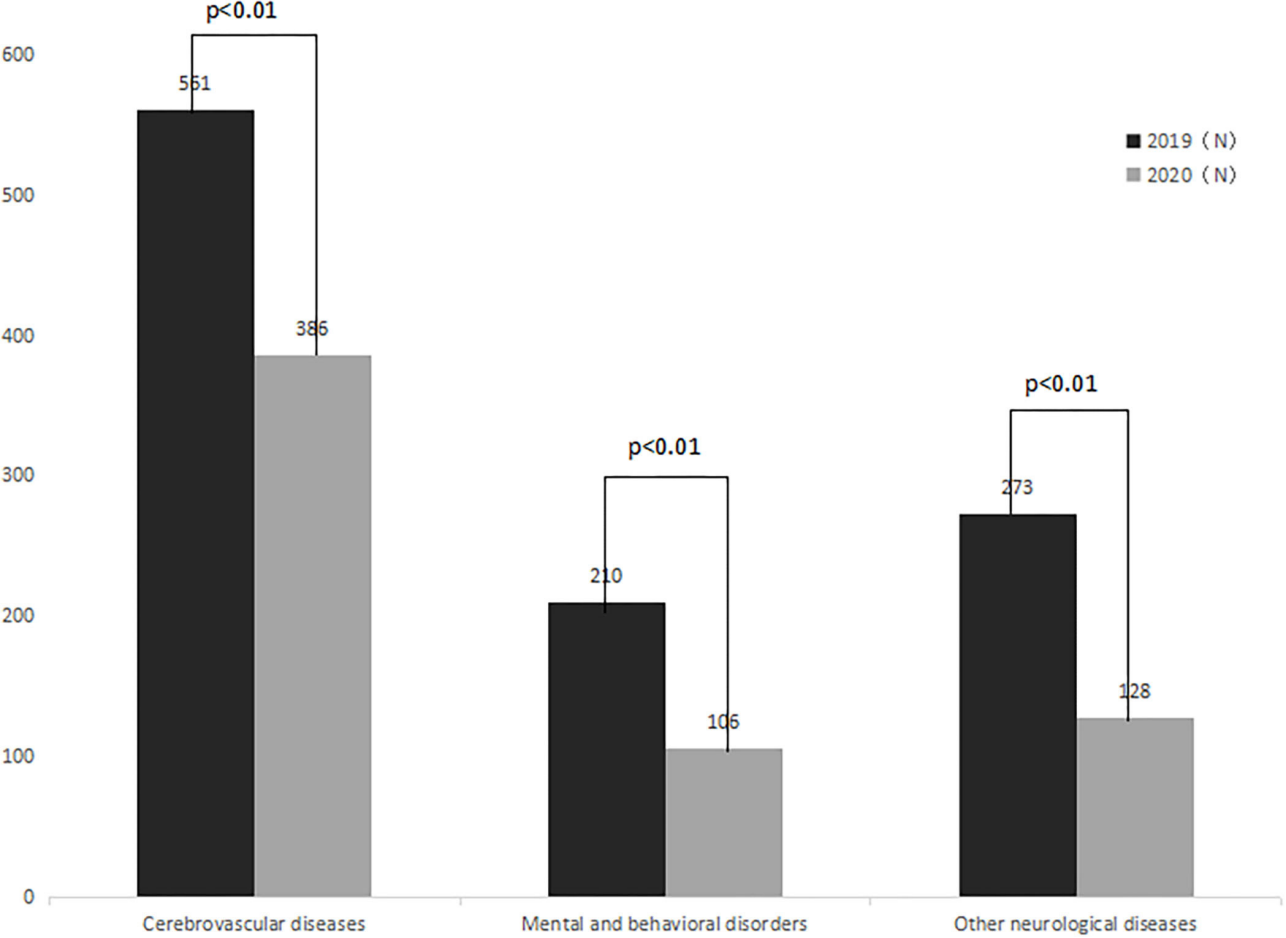
Comparison between the number of emergency calls for cerebrovascular diseases, mental and behavioral disorders, and other neurological diseases in 2019 and 2020.

From Figure 4, it can be seen that the number of emergency calls for almost every day in 2020 was reduced as compared to the number of calls for the same days in 2019. After January 23, the number of emergency calls decreased most significantly, and it then gradually increased until March 15, when the Hangzhou community lifted its closure restrictions. Figures 4, 5 reveal that, as compared with 2019, the number of ambulance dispatches and rescues dropped significantly after 23 January 2020. There was a short-term plateau from February 4 to February 10, and the numbers then continued to decline until March 15, when the number of ambulance dispatches and rescues began to converge with those values for the same period in 2019.

### Comparison between the number of emergency calls for cerebrovascular diseases, mental and behavioral disorders, and other neurological diseases in 2019 and 2020

A total of emergency calls for cerebrovascular diseases in the 2020 study period were 386, a decrease of 31.19% (95% CI: 24.32–33.81%) as compared with the same period in 2019 (*P* < 0.01), and a total of emergency calls for mental and behavioral disorders were 106, a decrease of 49.52% (95% CI: 38.22–55.31%) as compared with the same period in 2019 (*P* < 0.01). The number of emergency calls for other neurological diseases was 128, a decrease of 53.11% (95%CI: 48.72–61.04%) as compared with the same period in 2019 (*P* < 0.01). These data are presented in Figure 5.

## Discussion

The COVID-19 pandemic has drastically changed medical routines around the world. The available data do not confirm reductions in ambulance callouts for patients with stroke or heart attack in the UK (13). However, Valent et al. demonstrated that there was a decrease in the proportion of calls for neurologic symptoms from March to May 2020 in Italy (14). The results of this study suggest a decline in medical emergency calls and calls for CNS symptoms during the COVID-19 outbreak in Hangzhou City, China. The unknown cause of the decrease in the number of emergency calls inspired the present research. Data from the HEC were introduced, and several possible reasons for this phenomenon are subsequently provided. Compared with the same period in 2019, from 20 January to 20 March 2020, the total number of emergency calls and the number of emergency calls for CNS symptoms to the HEC decreased significantly. Although China's COVID-19 outbreak has been effectively controlled, and currently only small-scale infections have occurred in a few areas, the United States, Brazil, India, and other countries are still in the rising stage of the epidemic (15–17). A retrospective analysis of the reduction of emergency calls during the COVID-19 epidemic was conducted to provide a reference for other countries to reduce the impacts of COVID-19 on their own EMSs in China.

Based on the available data, it was found that the actual rate of ambulances was only about 30%, which means that an ambulance was not dispatched for the other 70% of the calls to the HEC. The reasons for this are as follows: (1) after considering the arrival time of the ambulance and the time it would take to get to the hospital by himself/herself, the caller chose to transport himself/herself to the hospital to receive medical assistance; (2) some callers tended to be in a very tense mood when calling the emergency number, which caused some meaningless or non-emergency calls as compared to calls for more serious medical emergencies; in these cases, the emergency dispatcher provided relevant medical guidance and instructed the caller to take himself/herself to a hospital if necessary; (3) for car accident patients, 110 and/or 119 (the emergency numbers for police and firefighters, respectively), were also called, thereby reducing the burden of the ambulance system to some extent, as these services are capable of dealing with some minor injuries, and the medical knowledge and means of transportation of these emergency dispatchers allowed them to relieve patient's pain and transport them to the hospital for treatment.

Based on the reduction in the number of emergency calls, the research results indicate that the COVID-19 outbreak had a certain degree of influence on the EMS in Hangzhou. However, it must be considered that due to the work schedule of Hangzhou at that time, respiratory symptoms, especially those of suspected patients with COVID-19, required special negative-pressure ambulances and professional staff for transport and emergency treatment. Thus, a substantial increase in the number of calls for respiratory illnesses was not found; instead, similar to other illnesses, the number of calls decreased. There are many possible reasons for the decline in emergency calls during the COVID-19 pandemic. First, considering that most work units and schools moved to online platforms and a home isolation policy was implemented, a possible explanation is a change in lifestyle. The reduction of commuting is intuitively related to lower anxiety and a lesser promotion of cerebrovascular accidents. Second, the people of Hangzhou City expressed doubts about whether the ambulance disinfection during the COVID-19 pandemic was adequate, and whether they would be infected with COVID-19 due to ambulance transportation. Therefore, this reduced the use of ambulances, and patients either used private cars to travel to the hospital or forewent going to the hospital altogether. Third, due to a fear of contracting COVID-19 exacerbated by media reports, people may have also been reluctant to go to the hospital for treatment. For many people with risk factors for cerebrovascular diseases, especially those with previous CNS symptoms, they or their family members may have believed that staying at home was a better choice than to risk of infection with COVID-19 at the hospital. The increased incidence of cerebrovascular accidents and aggravation of psychiatric symptoms in the later stage of the 2020 study period also support this explanation. In addition, the overwhelming impact of COVID-19 on the EMS may have caused medical staff to fail to recognize and diagnose CNS symptoms, thereby artificially reducing the incidence during this period.

In view of these reasons for the decline in emergency calls during the COVID-19 pandemic, the following solutions are proposed. First, the public should be informed that contact with COVID-19 within the EMS can be minimized, so they can continue to call the EMS to inform staff of CNS symptoms and, therefore, receive first aid and referral services. Second, for patients experiencing cerebrovascular accidents, especially patients with acute stroke, a green channel for diagnosis and treatment should be opened. Intravenous thrombolytic therapy and interventional therapy for artery occlusion should be carried out within a certain time window to shorten the treatment time, and medical staff should strive for better prognoses for patients with stroke. Third, it is proposed that a “one-key first aid” function can be added to the health code platform, e.g., the health code on the Alipay platform can be used to determine whether an individual has traveled to a high-risk area according to the trajectory of action. This is often used as the first step in the initial screening. The emergency center dispatch station can obtain the identity information and current location of the patient in real time after receiving the patient's help signal, and an ambulance can be quickly dispatched to the scene of the incident. Emergency doctors can then review the health files of the patient on the way to the scene to understand their medical history and health conditions and can, therefore, make more preparations for on-site precise treatment. For example, for patients with past mental and behavioral disorders, a specialist hospital can be contacted in advance to reduce the transfer time; for patients with symptoms and tendencies of cerebrovascular diseases, a nearby stroke center can be chosen to reduce the door-to-needle time (DNT) or the door-to-puncture time (DPT).

This research was characterized by certain shortcomings. First, this article only discussed the data from 2019 and 2020, and did not discuss the changes in data before 2019. Thus, it is impossible to clearly understand whether the decrease in emergency calls in 2020 was due to an existing long-term change trend in Hangzhou, or whether the impact of COVID-19 led to a reduction in the number of calls in 2020. Second, this research only focused on the city of Hangzhou in Zhejiang Province, China; whether the results obtained in this research can be generalized to the whole of China, and even to the world, remains worthy of future consideration and research. Third, the infection prevalence and fatality rate in Hangzhou during the COVID-19 epidemic were relatively low. Finally, we did not analyze each type of Emergency Medical Service in detail like Jaffe et al. (18). We only analyzed the emergency calls due to CNS symptoms. Therefore, the applicability of the results of this study to countries with high COVID-19 infection prevalence and fatality must be further explored.

In short, compared with the same period in 2019, it was found that the total number of emergency calls and the number of emergency calls for CNS symptoms during the COVID-19 epidemic in Hangzhou exhibited statistically significant decreases. However, this research was limited by the consideration of only the impact on a single emergency center, and Hangzhou is not a city with a higher COVID-19 infection rate. As of 15 March 2020, there were a total of 181 cases in the city, after which there were no more cases until August 1. In cities more affected by COVID-19, the findings of this study may be more apparent. Further research can evaluate how the number of emergency calls for CNS symptoms rebounded after the COVID-19 pandemic. At present, it can only be speculated that the number of emergency calls for CNS symptoms has significantly decreased during the COVID-19 pandemic.

## Conclusion

The total number of medical emergency calls and the number of emergency calls for CNS symptoms decreased significantly during the COVID-19 epidemic in Hangzhou, China. Through our preliminary analysis and discussion, we believe that we should take various measures to eliminate patients' concerns about medical treatment caused by the COVID-19 epidemic.

## Data availability statement

The original contributions presented in the study are included in the article/Supplementary material, further inquiries can be directed to the corresponding authors.

## Ethics statement

This study was approved by the Ethics Committee of Human Studies of the Affiliated Hospital of Hangzhou Normal University (2022(E2)-KY-108).

## Author contributions

PY and MW conceived the study and designed the analysis. YC, CC, and QW performed statistical analysis. ZF, JT, and WW wrote the first draft of the manuscript. MZ, XF, and XZ participate in revision the manuscript. All authors contributed to revision of the manuscript and approved the submitted version.

## Funding

This study was supported by the Hangzhou Science and Technology Bureau Fund (Grant Nos. 20191203B96 and 20191203B105), the Medical and Technology Project of Zhejiang Province (Grant Nos. 2020362651 and 2021KY890), the Clinical Research Fund of Zhejiang Medical Association (Grant No. 2020ZYC-A13), the Hangzhou Health and Family Planning Technology Plan Key Projects (Grant No. 2017ZD02), the Zhejiang Medical and Health Science and Technology Plan Project (Grant No. 2019RC245), the Hangzhou Agricultural and Social Development Research Active Design Project (Grant No. 20190101A03), and the Zhejiang Traditional Chinese Medicine Scientific Research Fund Project (Grant No. 2022ZB280). The study was also supported by the Key Medical Disciplines of Hangzhou. The funders have no role in the data collection, data analysis, preparation of manuscript, and decision to submission.

## Conflict of interest

The authors declare that the research was conducted in the absence of any commercial or financial relationships that could be construed as a potential conflict of interest.

## Publisher's note

All claims expressed in this article are solely those of the authors and do not necessarily represent those of their affiliated organizations, or those of the publisher, the editors and the reviewers. Any product that may be evaluated in this article, or claim that may be made by its manufacturer, is not guaranteed or endorsed by the publisher.
